# *Streptococcus lutetiensis* Induces Autophagy via Oxidative Stress in Bovine Mammary Epithelial Cells

**DOI:** 10.1155/2022/2549772

**Published:** 2022-02-07

**Authors:** Peng Chen, Jingyue Yang, Naiwen Wu, Bo Han, John P. Kastelic, Jian Gao

**Affiliations:** ^1^Department of Clinical Veterinary Medicine, College of Veterinary Medicine, China Agricultural University, Beijing 100193, China; ^2^Department of Production Animal Health, Faculty of Veterinary Medicine, University of Calgary, Calgary, AB, Canada T2N 4N1

## Abstract

*Streptococcus lutetiensis*, an emerging pathogen causing bovine mastitis, has not been well characterized. We reported that *S. lutetiensis* was pathogenic both *in vivo* and *in vitro* and caused inflammatory reactions in the mammary gland. However, roles of autophagy and oxidative stress in the pathogenesis of *S. lutetiensis*-induced mastitis are unclear. In this study, an autophagy model of *S. lutetiensis*-infected bovine mammary epithelial cells (bMECs) was used to assess oxidative stress and autophagy flux. Expressions of Beclin1, light chain 3II, and Sequestosome 1/p62 were elevated in bMECs after *S. lutetiensis* infection. In addition, autophagosome and lysosome formation confirmed autophagy occurred. Based on LysoTracker Red and acridine orange, lysosome degradation was blocked, and lower expressions of lysosomal-associated membrane protein 2, cathepsins D, and cathepsins L confirmed lysosomal damage. Concurrently, the nuclear factor erythroid 2-related factor 2 (Nrf2), kelch-like ECH-associated protein 1 (Keap1), heme oxygenase 1 (HO1), and NAD (P)H: quinone oxidoreductase 1 (NQO1), and basilic proteins associated with the Nrf2/Keap1 signaling pathway, were detected. Decreased keap1 and increased Nrf2, HO1, NQO1, and reactive oxygen species (ROS) indicated increased oxidative stress. Treatment with N-Acetyl-L-cysteine (NAC), an ROS inhibitor, decreased both oxidative stress and autophagy. Therefore, we concluded that *S. lutetiensis* caused intracellular oxidative stress and autophagy in bMECs. In addition, crosstalk between autophagy and oxidative stress affected the autophagic flux and blocked downstream autophagy. The Nrf2-keap1-p62 pathway participated in this process, with ROS acting upstream of these effects, interfering with normal cell functions.

## 1. Introduction

Mastitis is highly prevalent in dairy cows [1], causing huge losses. Bacterial infections, often *Streptococci* spp., are the most common cause of mastitis [2]. *S. lutetiensis*, increasingly isolated from milk (Chen et al., unpublished), caused mammary gland edema and hyperemia, and we reported that bovine-derived *S. lutetiensis* was often resistant to multiple antibiotics [3].

Autophagy is a catabolic pathway in eukaryotic cells to remove intracellular organelles under extreme environments, to degrade damaged organelles, and control intracellular bacterial infections [4]. There are three types: macroautophagy (also called autophagy), microautophagy, and chaperone-mediated autophagy [5]. Autophagy typically involves formation of autophagosomes, fusion between autophagosomes and lysosomes, and lysosomal degradation [6]. The term “autophagic flux” includes autophagosome synthesis, delivery of autophagic substrates into the lysosome, and their subsequent degradation inside the lysosome [7]. Groups A and B *Streptococci* induced autophagy [8, 9]; however, the ability of group D *Streptococci* to induce autophagy has apparently not been reported.

An autophagosome is a double-membrane vesicle involved in macroautophagy [10]. Lysosomes are monolayer-coated vesicles containing various acidic hydrolases [11] that degrade pathogens in autolysosomes. Beclin 1 and Atgs initiate autophagy and can form complexes of several proteins that regulate autophagosome maturation and transportation [12–14]. As an essential regulator, beclin 1 ubiquitination-regulated autophagy is important in inflammation [15]. In addition, lysosomal-associated membrane protein 2 (LAMP2) is a major component of the lysosomal membrane [16], promoting membrane integrity and fusion of autophagy vesicles and lysosomes [17]. Cathepsins D (CTSD) and cathepsins L (CTSL) are the most abundant lysosomal proteases [18]. Lysosomes need an acidic environment, with hydrolysis promoted by pH < 5.2 [11].

Reactive oxygen species (ROS) are highly reactive oxygen-containing substances. Although ROS concentrations are well regulated under physiologic conditions, if control mechanisms are disrupted, ROS concentrations can increase and may cause damage or disease. ROS is an early inducer of autophagy during nutrient deficiency [19] and can promote degradation of ubiquitinated materials [20].

The antioxidant transcription factor, Nrf2 (nuclear factor erythroid 2-related factor 2), is activated by p62 in a “noncanonical” pathway [21]. Nrf2 is an important transcription factor regulating cellular redox homeostasis. Under physiologic conditions, Nrf2 concentration is low, whereas under oxidative stress, it is activated and translocated from the cytoplasm into the nucleus [22]; this can trigger downstream target genes responsible for detoxification and elimination of harmful substances [23]. The protein expression level of Kelch-like ECH-associated protein 1 (keap1) is a negative regulator of Nrf2 [24]. There are indications of interrelations between Nrf2 and p62 and that Nrf2 has a role in dysregulation of autophagy [25].

We reported that *S. lutetiensis* adhered to, invaded, and destroyed bovine mammary epithelial cells (bMECs) and damaged murine mammary tissue [3]. However, roles of autophagy and oxidative stress in *S. lutetiensis*-induced mastitis are unclear. In this study, we used an autophagy model of *S. lutetiensis*-infected bMECs to assess oxidative stress and autophagy flux.

## 2. Materials and Methods

### 2.1. Reagents and Antibodies

Acridine orange (AO) was from Solarbio. N-Acetyl-L-cysteine (NAC) was from Sigma. Bicinchoninic acid (BCA) protein assay kit and enhanced chemiluminescence kit were from Cwbio. RIPA buffer and LysoTracker Deep Red were from Beyotime Biotechnology. For these compounds, catalog information is listed in Table 1. Western blotting and immunofluorescence staining used the following primary antibodies: anti-CTSL/major excreted protein and anti-CTSD from ABclonal Technology; anti-light chain 3 (LC3) B from Beyotime (Shanghai, China); anti-Sequestosome 1/p62 (SQSTM1/p62), anti-glyceraldehyde 3-phosphate dehydrogenase (GAPDH), anti-lysosome-associated membrane protein 2 (LAMP2), anti-*β*-actin, anti-*α*-tubulin, and anti-Beclin 1, from Protein tech; and Peroxidase-Conjugated AffiniPure Goat Anti-mouse IgG and goat anti-rabbit IgG from Cwbio. Catalog and dilution information of these reagents are listed in Table 2.

### 2.2. Bacterial Strain and Cell Culture

*S. lutetiensis* strain was cultured in Brain Heart Infusion (BHI) broth at 37°C for 12 h. After reaching OD_600_ = 0.8‐1.2, bacteria were washed three times with Dulbecco's modified Eagle medium (DMEM) without serum. The bovine mammary epithelial cell line (MAC-T) was digested with trypsin for 2 min at 37°C and centrifuged at 100 × g for 5 min. The bMECs were cultured overnight at 37°C in 5% CO_2_ without antibiotics in DMEM, supplemented with 10% (*v*/*v*) heat-inactivated fetal bovine serum (FBS) until cell density reached 80%.

### 2.3. CCK-8 Assay

MAC-T cells (~4,000 cells/well) were inoculated into 96-well plates. After being infected with *S. lutetiensis* (MOI = 100), a CCK-8 kit was used to evaluate cell viability, in accordance with label protocols. After infection, 10 *μ*l of CCK-8 solution was added in each well and incubated at 37°C for 2 h; finally, the absorbance was read at 490 nm (680 Multipurpose Microplate Reader, Bio-Rad Laboratories).

### 2.4. Infection of bMECs with *S. lutetiensis*

**Figure 1:**
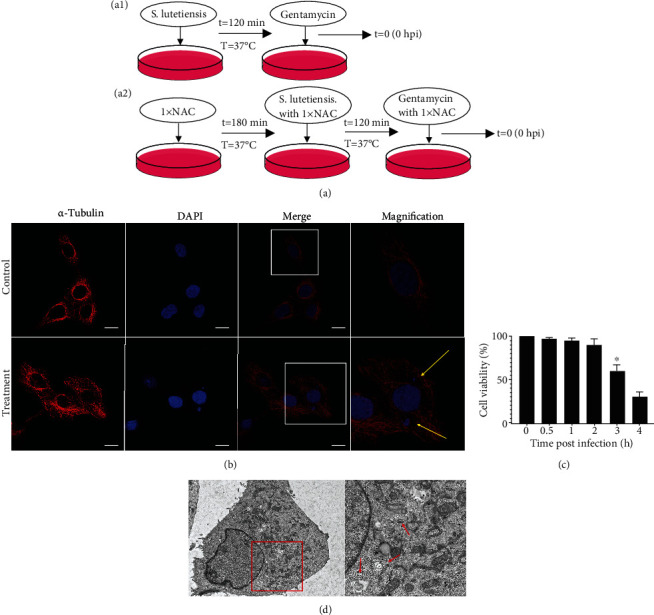
Our infection model of MAC-T cells was successful. (a) Schematic representation of our experimental design. (a1) MAC-T cells were incubated with *S. lutetiensis* for 120 min at 37°C; thereafter, extracellular bacteria were killed with gentamycin. Recording of the experimental process started with time point 0 h postinfection (hpi). (a2) MAC-T cells were incubated with 1× NAC for 180 min at 37°C, then incubated with *S. lutetiensis* for 120 min at 37°C. Afterwards, all extracellular bacteria were killed with gentamycin. Recording of the experimental process started with time point 0 h postinfection (hpi). (b) Representative confocal images of invading *S. lutetiensis* and localization of tubulin with DAPI. Magnification of the outlined area, showing details; “→” points to *S. lutetiensis.* Scale bars: 10 *μ*m. (c) Effects of *S. lutetiensis* invasion time on cell activity. Values are mean ± SD, *n* = 3, ^∗^*p* < 0.05. (d) Transmission electron microscopy. Infected for 2 h with *S. lutetiensis*; “→” points to *S. lutetiensis* inside a double-layer membrane. Scale bars: 20 *μ*m.

Autophagy caused by intracellular *S. lutetiensis* in bMECs was explored. Our intracellular infection model was modified from a previous study [24]. In the present study, bMECs seeded at 2 × 10^5^ cells/well in six-well plates were infected with fresh *S. lutetiensis* grown with BHI broth, at multiplicity of infection (MOI) of 100. Cells were incubated for 2 h at 37°C in 5% CO_2_ to allow bacterial uptake and invasion, then washed three times with phosphate-buffered saline (PBS) and DMEM/10% FBS with 100 *μ*g/ml gentamycin (bacterial inhibiting buffer) added into each well to kill extracellular bacteria. After changing the medium to DMEM/10% FBS with 100 *μ*g/ml gentamicin, cells were incubated for the indicated intervals at 37°C in 5% CO_2_. If not stated otherwise, inhibitors were added to incubation buffer (Figure 1(a)). To confirm the role of oxidative stress, NAC was used to pretreat cells for 3 h, followed by exposure to *S. lutetiensis* (MOI = 100) for 2 h and, finally, incubation in medium with 100 *μ*g/ml gentamycin. The bMECs were incubated in 1× NAC from the start of NAC pretreatment to cell harvest (Figure 1(a)).

### 2.5. Western Blotting

For Western blot analysis, after being infected with *S. lutetiensis* for defined intervals, MAC-T cells were collected and lysed with 200 *μ*l RIPA buffer (with 1% phenylmethylsulfonyl fluoride) for 10 min on an ice-plate, then centrifuged at 12,000 × g for 15 min and protein concentrations quantified with a BCA protein quantity reagent kit. Equal volumes of lysates were separated by sodium dodecyl sulphate-polyacrylamide gel electrophoresis and transferred to a polyvinylidene difluoride membrane by Wet blotting. Blots were blocked in Tris-buffered saline (TBS) containing 5% skim milk powder. Membranes were incubated with primary antibodies (Beclin 1, LC3 II/I, p62, CTSD are referred to GAPDH, and LAMP2, CTSL, Nrf2, Keap1, HO-1, and NQO-1 are referred to *α*-tubulin) at 4°C overnight, washed with TBS buffer with Tween 20, then incubated with secondary antibodies for 1 h at RT. Blotting signals were detected with an enhanced chemiluminescence Western blot detection system, images collected, and relative band density analyzed with Image J software (NIH, Bethesda, MD, USA).

### 2.6. Enzyme-Linked Immunosorbent Assay (ELISA)

The superoxide dismutase (SOD) (Catalog: ml036559, mlbio, Shanghai, China), malondialdehyde (MDA) (Catalog: ml402351, mlbio), and glutathione (GSH) (Catalog: ml063357, mlbio) contents in the proteins of MAC-T cells were measured with an ELISA kit (mlbio), in accordance with label instructions.

### 2.7. Transient Transfection

Autophagy flux was confirmed by analyzing formation of fluorescent puncta of autophagosomes and lysosomes in GFP-LC3- or mCherry-GFP-LC3-transfected cells. In this study, MAC-T cells were transfected with GFP-LC3 or mCherry-GFP-LC3 adenovirus, according to label protocols. After treatment, cells were fixed with 4% paraformaldehyde for 10 min and dyed with Hoechst 33342 for 5 min at RT. Confocal microscopy was used to examine transfected cells, and representative cells were photographed.

### 2.8. Transmission Electron Microscopy

To assess ultrastructure, cells were seeded on six-well plates infected with *S. lutetiensis* as described above, fixed in 4% paraformaldehyde in PBS overnight at RT, postfixed in 2% osmium tetroxide, dehydrated in a graded ethanol series (50, 70, 80, 90, and 100% for 15 min each), and embedded in epoxy resin. Finally, they were kept at 37°C for 12 h and 45°C for 24 h and, finally, cured at 60°C for 24 h. Ultrathin sections were prepared, stained, and examined with a Zeiss TEM 910 (Zeiss, Oberkochen, Germany).

### 2.9. Acridine Orange (AO) and LysoTracker Red (LTR) Staining

Cells were seeded on glass coverslips in six-well plates infected with *S. lutetiensis*, as described above. Cells were incubated in 5 *μ*g/ml AO or 100 nM LTR at 37°C for 30 min and fluorescence signals detected with confocal microscopy.

### 2.10. Measurement of Reactive Oxygen Species (ROS) Levels

Intracellular ROS generation was monitored using a fluorescence probe dichloro-dihydro-fluorescein diacetate (DCFH-DA). After treatment, cells were collected, washed three times with PBS, and incubated in darkness with 100 *μ*M DCFH-DA for 30 min at 37°C. Cells were harvested, resuspended in PBS, and filtered through a 70 *μ*m filter. Flow cytometry was used to measure ROS concentrations, based on fluorescence intensity (FL-1, 530 nm) of 10,000 cells. Meanwhile, the samples were detected with a fluorometric reader Spectra Max i3x (MD, America) at 488 nm excitation wavelength and 525 nm emission wavelength.

### 2.11. Immunofluorescence Staining

Cells were seeded on sterile coverslips placed in 6-well plates. After cells were infected with *S. lutetiensis* for 2 h, they were fixed in 4% paraformaldehyde for 10 min and then permeabilized with 0.2% Triton X-114 in PBS for 15 min. After being washed with PBS, cells were blocked with 3% BSA for 1 h at RT. Slides were incubated with anti-*α*-tubulin antibody (1 : 200 diluted in PBS) overnight at 4°C and then incubated with peroxidase-conjugated AffiniPure (diluted 1 : 100 in PBS) second antibody for 1 h at 37°C, before nuclei and intracellular bacteria were stained with DAPI. Between steps, cells were thrice-washed with PBS. Ultimately, all slides were mounted with antifade mounting medium and examined on a Nikon A1HD25 confocal microscope with a 100x oil immersion objective. Imaging used laser wavelengths of 488 and 561 nm.

### 2.12. Statistical Analyses

All statistical analyses were performed with Statistical Product and Service Solutions 19.0 software (SPSS Inc., Chicago, IL, USA). One-way ANOVA was used to determine effects of group and time, and Duncan's multiple range test was used to locate significant differences. For all statistical analyses, *p* < 0.05 was considered significant. Results are expressed as mean ± standard deviation (SD). Data were displayed with GraphPad Prism V8.0 (Data Analysis and Graphing Software, San Diego, CA, USA).

## 3. Results

### 3.1. Successful Cell Infection Model

*S. lutetiensis* was observed in MAC-T cells of the treated group but was absent in the control group (Figure 1(b)). Based on the CCK-8 assay, continuous infection with *S. lutetiensis* at MOI = 100 for 2 h did not decrease cell activity (Figure 1(c)). However, in ultrastructure images, *S. lutetiensis* was present in MAC-T cells, and there was an autophagic vesicle membrane structure around the bacteria (Figure 1(d)).

### 3.2. Intracellular *S. lutetiensis*-Induced Autophagy

**Figure 2:**
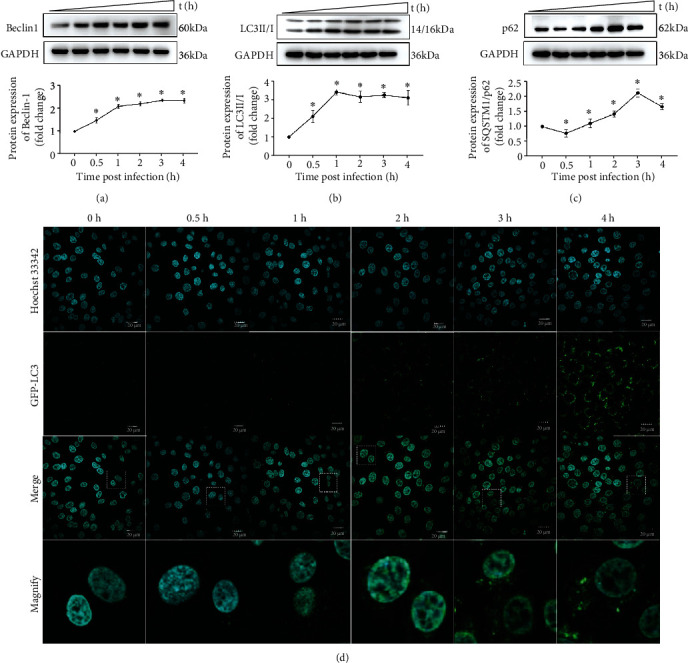
Intracellular *S. lutetiensis*-induced autophagy. (a–c) Protein level of Beclin1, LC3, and p62 in MAC-T cells with various treatments, with quantitative analysis under Western blotting bands. Data are mean ± SD, *n* = 3, ^∗^ represents significance with the 0 h group, ^∗^*p* < 0.05. (d) Formation of GFP-LC3 puncta was observed with confocal microscopy. Scale bars: 20 *μ*m.

Infection with *S. lutetiensis* substantially increased expression of Beclin 1 (Figure 2(a)). Furthermore, in bMECs treated with *S. lutetiensis*, conversion of LC3I to LC3II increased significantly (Figure 2(b)), starting from the 1^st^ hour postinfection (hpi) and remaining elevated for the following 3 h, when a green fluorescent protein (GFP)-LC3 labeled autophagosome was detected by confocal imagine (Figure 2(d)). Autophagosomes (fluorescent spots) increased with interval after infection. The protein expression level of SQSTM1/p62 tended to decrease at 0.5 hpi, and then increased from 1 to 3 hpi (Figure 2(c)).

### 3.3. Autophagy Flux Disorder

**Figure 3:**
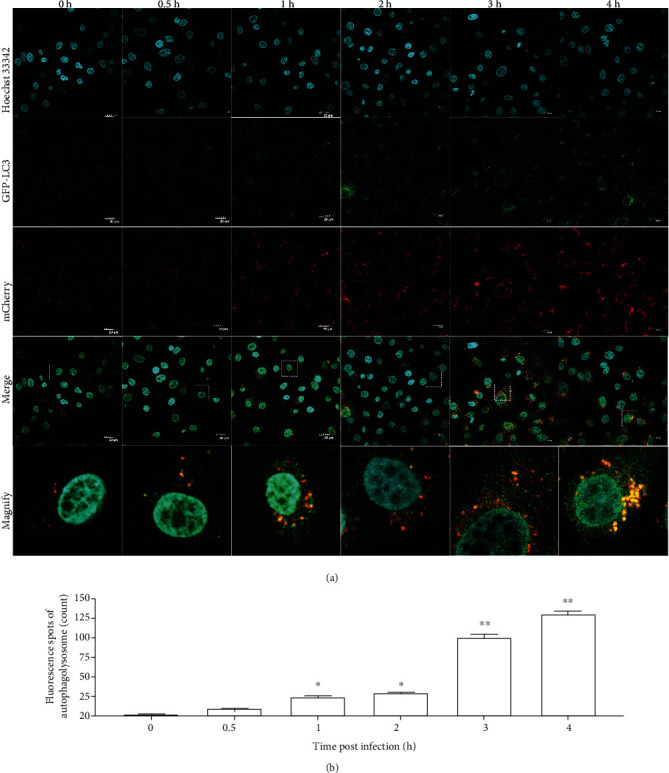
Autophagy flux disorder. Cells were grown on coverslips and transfected with mCherry-GFP-LC3 at MOI = 20 for 24 h, then infected with *S. lutetiensis* for various intervals to monitor the autophagic flux; (a) representative confocal images. Scale bars: 20 *μ*m. (b) Quantity of autophagolysosome (mean ± SD, *n* = 3, ^∗^ represents the significance between adjacent time points, ^∗^*p* < 0.05, ^∗∗^*p* < 0.01).

To determine whether the autophagy flux was blocked after infection with *S. lutetiensis*, mCherry-GFP-LC3, which can label lysosomes, was transfected into MAC-T cells (Figure 3). Yellow fluorescence (green fluorescence merged with red fluorescence) became increasingly prominent during the postinfection period, with red mottled fluorescence peaking at 3 hpi (Figure 3(a)), and yellow fluorescence spots quantified (Figure 3(b)).

### 3.4. *S. lutetiensis* Reduced Lysosomal pH

**Figure 4:**
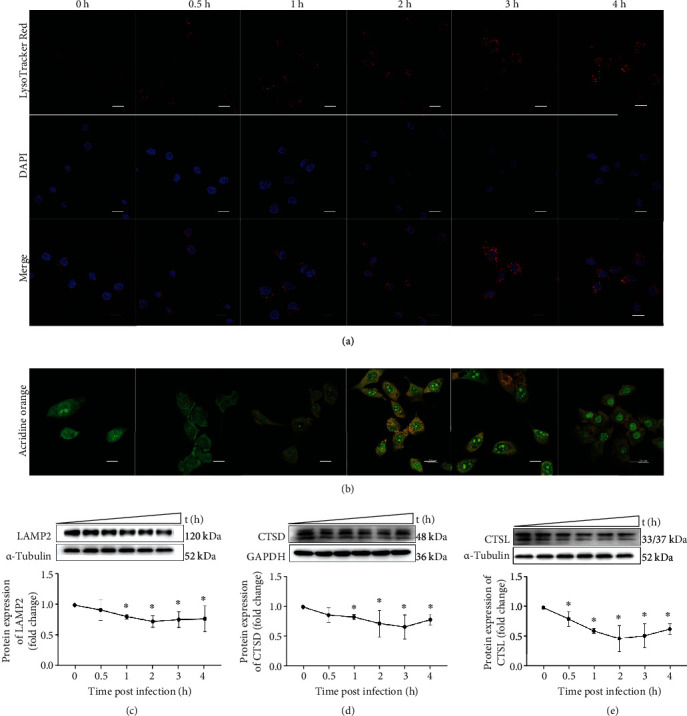
*S. lutetiensis* decreased pH in lysosomes to further block the autophagy flux. (a) To assess lysosomal pH, cells were stained with 100 nM LysoTracker Deep Red at 37°C for 30 min after being infected with *S. lutetiensis*. Scale bars: 20 *μ*m. (b) After being infected with *S. lutetiensis*, cells were stained with AO at 37°C to assess lysosomal pH. Scale bars: 20 *μ*m. (c–e) Protein levels of LAMP2, cathepsin D, and cathepsin L in MAC-T cells at various intervals after infection with *S. lutetiensis*. Upper panels: representative Western blot images; lower panels: quantitative analysis (mean ± SD, *n* = 3, ^∗^ represents the significance with the 0 h group, ^∗^*p* < 0.05).

Infection with *S. lutetiensis* increased LTR staining, consistent with lysosomal acidification (Figures 4(a) and 4(b)). Red spots increased over time and accumulated around the nucleus, from 0 to 3 h, with a modest decline at 4 h. After staining with AO, the color changed over time. They changed from 1 h, peaking at 3 h, and a slight decrease at 4 h.

### 3.5. Degradation of Impaired Lysosomes

During the final stages of autophagy, autophagosomes fuse with lysosomes and are subsequently degraded. Thus, LAMP2 protein, detected via Western blotting, decreased over time (Figure 4(c)). Lysosome-related proteins CTSD and CTSL were also detected (Figures 4(d) and 4(e)). All three kinds of lysosome-associated proteins decreased over 3 hpi, indicating lysosomal degradation was impaired after *S. lutetiensis* infection of MAC-T.

### 3.6. *S. lutetiensis* Activated the Nrf2-Keap 1 Pathway in MAC-T Cells

**Figure 5:**
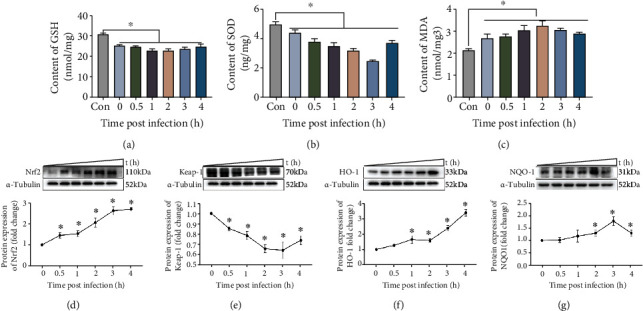
*S. lutetiensis* induce oxidative stress in MAC-T cells. (a–c) Protein levels of GSH, SOD, and MDA detected by ELISA. (d–g) Protein levels of Nrf2, keap1, HO-1, and NQO-1 in MAC-T cells at various intervals after infection with *S. lutetiensis*. Upper panels: representative Western blot images; lower panels: quantitative analysis (mean ± SD, *n* = 3, ^∗^ represents the significance with 0 h group, ^∗^*p* < 0.05).

Infection with *S. lutetiensis* decreased GSH and SOD (Figures 5(a) and 5(b)), but increased MDA (Figure 5(c)), essential indicators of oxidative stress. Regarding protein levels of Nrf2, keap1, NQO1, and HO1, *S. lutetiensis* increased Nrf2 protein level, whereas the Keap1 protein level was significantly below baseline (Figures 5(d) and 5(e)). HO-1 and NQO-1 are detoxifying enzymes with important roles in Nrf2-regulated phase II; protein levels of both were elevated over time in *S. lutetiensis*-infected cells (Figures 5(f) and 5(g)). Between 1 and 2 h after infection, HO-1 had a transitory decrease, whereas NQO-1 had a slight decrease at 4 h. Therefore, the Nrf2-keap1 pathway was active when *S. lutetiensis* invaded MAC-T cells and induced oxidative stress.

### 3.7. ROS Contributed to *S. lutetiensis*-Induced Autophagy in bMECs

**Figure 6:**
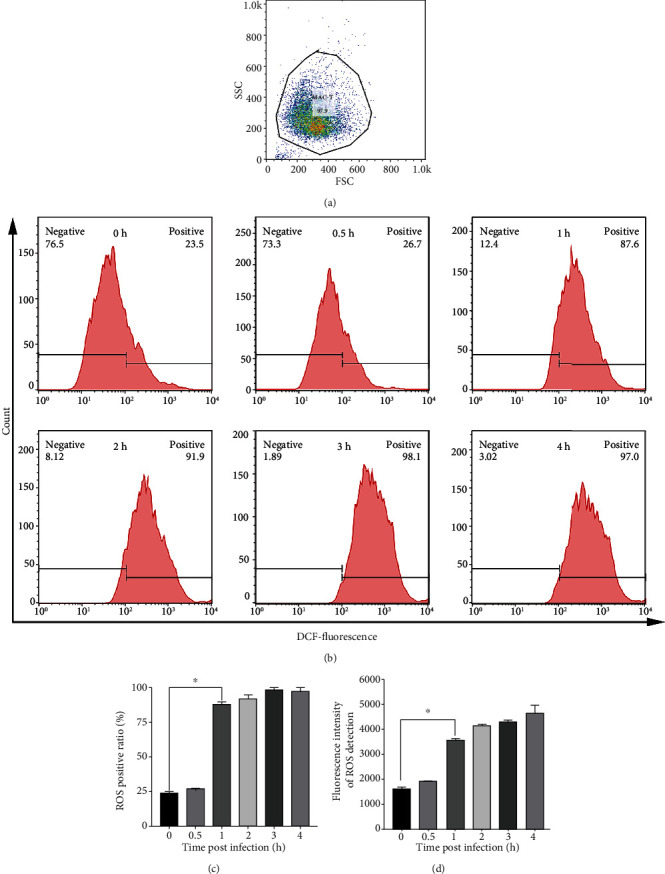
ROS expression in MAC-T cells at various intervals after infection with *S. lutetiensis* (a–c) ROS detected by flow cytometry. (d) Fluorescence intensity of ROS detected by fluorometric reader (mean ± SD, *n* = 3, ^∗^ represents the significance with 0 h group, ^∗^*p* < 0.05).

**Figure 7:**
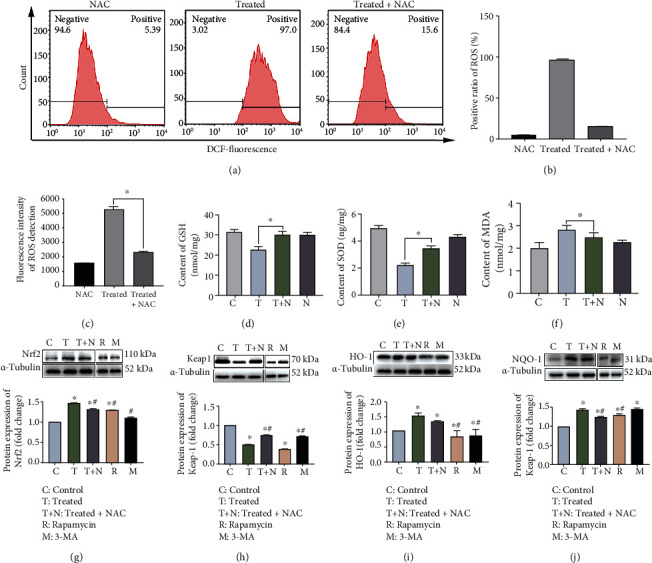
NAC efficiently decreased *S. lutetiensis*-induced oxidative stress in MAC-T. (a–c) The difference of ROS expression in MAC-T cells between treated with *S. lutetiensis* and treated with NAC (detected by flow cytometry and fluorometric reader). (d–f) Protein levels of GSH, SOD, and MDA detected by ELISA. (g–j) Protein levels of Nrf2, keap1, HO-1, and NQO-1 in MAC-T cells after various treatments. Upper panels: representative Western blot images; lower panels: quantitative analysis (mean ± SD, *n* = 3, ^∗^ represents the significance with the control group, ^#^ represents the significance with the treated group, ^∗^*p* < 0.05, ^#^*p* < 0.05; C: control; T: treatment; T+N: treatment + NAC; R: rapamycin; M: 3-MA).

**Figure 8:**
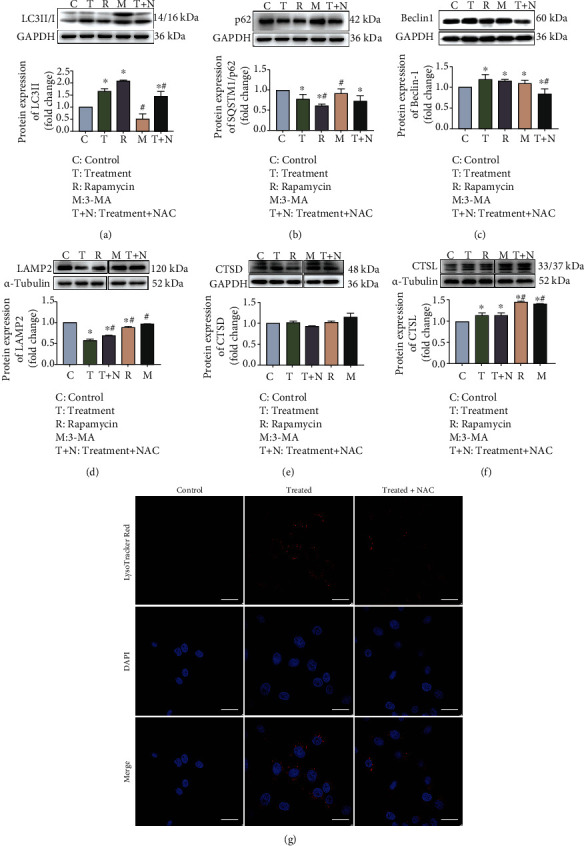
NAC efficiently decreased *S. lutetiensis*-induced autophagy in MAC-T. (a–f) Protein levels of LC3II/I, p62, Beclin1, LAMP2, CTSD, and CTSL. Upper panels: representative Western blot images; lower panels: quantitative analysis (mean ± SD, *n* = 3, ^∗^ represents the significance with the control group, ^#^ represents the significance with the treated group, ^∗^*p* < 0.05, ^#^*p* < 0.05; C: control; T: treatment; R: rapamycin; M: 3-MA; T+N: treatment + NAC). (g) Lysosome detection comparison between infected with *S. lutetiensis* with/without NAC. Scale bars: 20 *μ*m.

Oxidative stress is involved in *S. lutetiensis*-induced autophagy in MAC-T cells. To detect a link between ROS production and oxidative stress, ROS concentrations were assessed. Both flow cytometry analysis (Figures 6(a)–6(c)) and fluorometric reader detection (Figure 6(d)) indicated that *S. lutetiensis* increased oxidative stress and triggered excessive ROS in MAC-T cells, with increased generation of ROS infection. NAC, an inhibitor of ROS, was applied to determine whether oxidative stress was implicated in inhibition of autophagosome-lysosome fusion. Treatment with NAC significantly decreased ROS in cells infected with *S. lutetiensis* (Figures 7(a)–7(c)). In addition, treatment for 2 h with *S. lutetiensis* (MOI = 100) induced a time-dependent increase in ROS production, although the effect was abolished at 4 h (Figure 6(b)). In addition, NAC abolished ROS production (Figures 7(a)–7(c)) and consequently oxidative stress (Figures 7(d)–7(f)), with reductions in extent of changes in GSH, SOD, and MDA. Furthermore, Nrf2, HO-1, and NQO-1 were decreased significantly compared to the treated group, whereas Keap1 protein was recovered (Figures 7(g)–7(j)). We next assessed whether ROS was involved in *S. lutetiensis*-induced autophagy. *S. lutetiensis* increased Beclin 1 protein level in bMECs, as well as the LC3 II/I ratio and p62 degradation, whereas NAC prevented these effects (Figures 8(a)–8(c)). The lysosomal protein LAMP2 was decreased after infection, but after NAC, was increased compared to the control group (Figure 8(d)). However, neither CTSD nor CTSL expression was significantly different between the treatment group and NAC-supplemented group (Figures 8(e) and 8(f)). In addition, LTR fluorescence was used to examine autophagosome-lysosome fusion (Figure 8(g)). These results demonstrated that increased ROS production was an upstream event contributing to *S. lutetiensis* activation of autophagy.

## 4. Discussion

**Figure 9:**
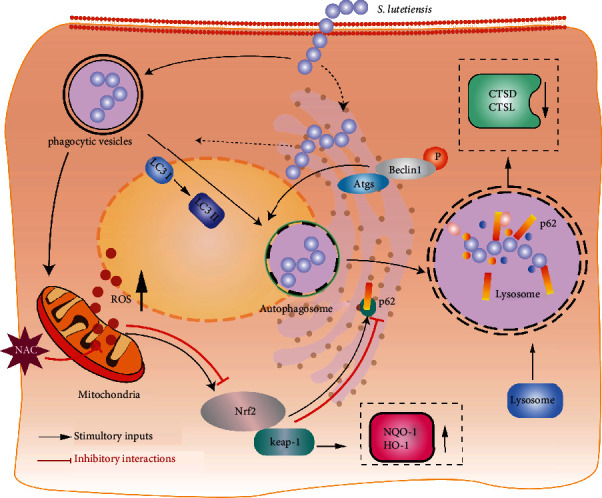
Putative signal pathway of *S. lutetiensis* inducing autophagy in bovine mammary gland epithelia though oxidative stress. Schematic diagram illustrating that *S. lutetiensis* can invade MAC-T cells and lead to autophagy by stimulating mitochondrial oxidative stress. In this process, the ROS-Nrf2-keap1 pathway has an essential role. ROS is upstream of these effects. Black arrows and red bars indicate stimulation and inhibition, respectively.

*S. lutetiensis* invades cells to evade immune defenses and survives within those cells [3]. Herein, the ability to induce autophagy was achieved following optimization of *S. lutetiensis* dose in MAC-T cells, without compromising cell viability. This model provided clear evidence that *S. lutetiensis* induced autophagy in MAC-T cells by increasing oxidative stress and that there was a close relationship between oxidative stress and autophagy. Furthermore, these findings provided insights into potential mechanisms underlying *S. lutetiensis*-induced autophagy (Figure 9).

In this study, *S. lutetiensis* was regarded as a repelling substance; the classical vesicles of autophagosome compartments were observed and engulfed by an autophagosome, typical characteristics indicating the host activated its defense mechanism, including initiation of autophagy [26]. Moreover, Beclin 1, which is essential for both autophagy and lysosomal enzyme transport [27], was increased after infection with *S. lutetiensis*. Furthermore, LC3 II, an autophagy marker with levels related to the intensity of autophagy [28], uses GFP-LC3 for confirming autophagy accumulation [29]. Therefore, expression of LC3 II was assessed by GFP-LC3 detected with confocal microscopy after MAC-T cells were infected with *S. lutetiensis*, with a time-independent tendency for green spots. Collectively, all of these outcomes indicated that *S. lutetiensis* induced autophagy.

SQSTM1/p62 acts as a relative protein to mediate degradation of its recognition substrate [30, 31]. Excessive build-up of SQSTM1/p62 sequestered keap1, an adaptor of the E3-ubiquitin ligase complex for Nrf2 [32]. In the present study, expression of SQSTM1/p62 protein began to rise from the second time point (0.5 h after infection) during *S. lutetiensis* infection in bMECs. Meanwhile, expression of Nrf2 increased, whereas keap1 decreased from 0 to 3 hpi, consistent with previous studies [24]. The mCherry-GFP-LC3 is an adenovirus probe to detect the rate of autophagic flux [33]. In this study, accumulation of yellow fluorescence was observed by mCherry-GFP-LC3 after *S. lutetiensis* infection, indicating that the autophagic flux was blocked at fusion of autophagosomes and lysosomes. In addition, *S. lutetiensis* induced increases in the LC3 II/I ratio and p62 degradation, providing further support for autophagy induction. Moreover, LAMP2 labelling, as well as cell staining with AO and LTR, demonstrated that *S. lutetiensis* weakened lysosomal activity.

Autophagy formation is the first step of autophagy flux. In this step, autophagosomes can fuse with lysosomes or late endosomes and then fuse with lysosomes [34]. Lysosomal degradation is an essential factor for autophagy flux, and a slightly acidic environment for lysosomes promotes proteolytic enzyme degradation of organelles [35]. LTR is a reliable probe to detect changes in intercellular pH, as it accumulates in an acidic environment, with the intensity of red fluorescence corresponding to the pH [36, 37]. In addition, AO is a specific probe that can be trapped in lysosomes [38]. In the present study, the intensity of LTR peaked at 3 h after *S. lutetiensis* infection, with AO staining having a similar outcome. These two approaches both provided evidence that lysosomes accumulated due to a blockage of the downstream autophagy flux, which differed from a report that group A *Streptococcus* was efficiently killed within a lysosome-fused autophagosome compartment [9].

Cathepsins are the major lysosomal proteases involved in autophagic degradation, wherein CTSD and CTSL are two abundant lysosomal proteases [39]. When activation of cathepsin proteases requires acidification, the altered pH decreased protein degradation [40]. CTSD is involved in intracellular catabolism in lysosomal compartments [41]; by activating CTSB, CTSL can hydrolyze proteins, hormones, and phagocytic bacteria [42]. In this study, expression of CTSD and CTSL decreased initially, but subsequently recovered to a basal level, indicating that intracellular invasion of *S. lutetiensis* had a negative effect on lysosomes, and cathepsins had self-healing ability.

As an essential mechanism for eliminating damaged organelles and exogenous foreign body, autophagy is regulated by various cellular process such as nutrient deficiencies, oxidative stress, and other influencing factors [43]. Autophagy and oxidative stress in a *S. lutetiensis*-infected model have apparently not been reported. Herein, we determined that oxidative stress had an essential role in *S. lutetiensis*-induced cyto-injury. As a transcription factor, Nrf2 regulates a cluster of oxidative stress-inducible genes in cells. There is considerable evidence that the Nrf2-Keap1 antioxidant system has an important cytoprotective defense mechanism in alleviating oxidative insults [44, 45]. Meanwhile, Keap1 can indirectly assess activation of Nrf2, as the Nrf2-Keap1 pathway is a regulator for the endogenous antioxidant response [46]. Therefore, Nrf2-Keap1 system integrity is maintained by an autophagy pathway [47], and activation of Nrf2 is prolonged if autophagy is disrupted [25]. In response to oxidative stress, Nrf2 transcription factor could induce expression of p62 [48, 49]. Our study implied that transportation of oxidized proteins to autophagosomes for degradation can decrease oxidative injury. In response to oxidative stimuli, keap1 lost its ability to bind with Nrf2. Therefore, we speculate that Nrf2 may enter the nucleus and bind to the antioxidant response element (ARE), to further regulate downstream genes HO-1 and NQO-1, as they are key oxidative stress indicators regulated by Nrf2 [24]. Furthermore, HO-1 and NQO-1 proteins in MAC-T cells were elevated after *S. lutetiensis* infection. Recent research demonstrated that NAC could block ROS production and further reversed LC3 II accumulation which triggered by coenzyme Q_0_, and the same mechanism of NAC also occurred in chrysin-induced autophagy [50, 51]. We used NAC, an efficient ROS inhibitor [52, 53]. Modulation of antioxidant enzymes (SOD, GSH, and MDA) indicated inhibition of oxidative stress. Meanwhile, expression of NQO-1 and HO-1 was restored to basal levels; therefore, we inferred that Nrf2 may be a key cytokine in oxidative stress and autophagy.

As arguably the most influential intracellular signaling molecule, ROS regulates cell function and promotes homeostasis [54]. There are indications that oxidative stress can be a vital stimulus, via regulation of ROS, to stimulate autophagy [21, 55]. Autophagy and oxidative stress have an intricate relationship in many diseases [26]; hyperthermia can enhance both autophagy and ROS generation, implying potential associations between autophagy and oxidative stress [56]. Moreover, several studies reported the relationship between oxidative damage and autophagy [57], although this has apparently not been reported in bMECs. In this study, ROS were rapidly produced after exposure to *S. lutetiensis*. Notably, after 2 h, *S. lutetiensis* induced accumulation of LC3II and degradation of p62, suggesting that ROS generation preceded initiation of autophagy. Furthermore, NAC effectively blocked autophagy activation by *S. lutetiensis*. As a main component of oxidative stress, ROS could be the link between autophagy and oxidative stress in bMECs.

The current study, apparently the first investigation of the role between autophagy and oxidative stress induced by *S. lutetiensis*, identified crosstalk between autophagy and oxidative stress. The limitation of this study was that an in vitro bMEC infection model was used to evaluate autophagy and oxidative stress. In future studies, we plan to investigate whether similar interactions occur in vivo in mammary tissue.

## 5. Conclusions

We characterized autophagy induced by oxidative stress in bMECs infected with *S. lutetiensis* isolated from bovine mastitis. We concluded that *S. lutetiensis* induced autophagy of bMECs by upregulating oxidative stress, although lysosomes accumulated due to a blockage of the downstream autophagy flux. In this process, there was crosstalk between autophagy and oxidative stress; the latter affected autophagy by intervening in the Nrf2-keap1-p62 pathway, with ROS acting upstream of these effects.

## Figures and Tables

**Table 1:** Reagent details.

Reagent	Catalog no.	Source	City
Acridine orange	A8120	Solarbio	Beijing, China
N-acetyl-L-cysteine	A7250	Sigma	St. Louis, MO, USA
Bicinchoninic acid (BCA) protein assay kit	CW0014S	Cwbio	Beijing, China
Enhanced chemiluminescence kit	CW0049S	Cwbio	Beijing, China
Ad-GFP-LC3B	C3006	Beyotime	Shanghai, China
Ad-mCherry-GFP-LC3B	C3011	Beyotime	Shanghai, China
LysoTracker Red	C1046	Beyotime	Shanghai, China
CCK-8	CK04	Dojindo	Kumamoto, Japan

**Table 2:** Antibody details.

Antibody	Catalog no.	Source	City	Dilution ratio
Anti-CTSD	A13292	ABclonal Technology	Wuhan, China	1 : 1000
Anti-CTSL	A12066	ABclonal Technology	Wuhan, China	1 : 1000
Anti-LC3B	AL221	Beyotime	Shanghai, China	1 : 1000
Anti-SQSTM1/p62	18420-1-AP	Proteintech	Wuhan, China	1 : 1000
Anti-GAPDH	60004-1-Ig	Proteintech	Wuhan, China	1 : 1000
Anti-LAMP2	AF1036	Beyotime	Shanghai, China	1 : 1000
Anti-*β*-actin	AA128	Beyotime	Shanghai, China	1 : 2000
Anti-*α*-tubulin	66031-1-Ig	Proteintech	Wuhan, China	1 : 2000
Anti-Beclin 1	11306-1-AP	Proteintech	Wuhan, China	1 : 2000
Anti-Nrf2	16396-1-AP	Proteintech	Wuhan, China	1 : 1000
Anti-keap1	10503-2-AP	Proteintech	Wuhan, China	1 : 1000
Anti-NQO1	11451-1-AP	Proteintech	Wuhan, China	1 : 1000
Anti-HO1	10701-1-AP	Proteintech	Wuhan, China	1 : 1000
Goat anti-mouse IgG	CW0102S	Cwbio	Beijing, China	1 : 3000
Goat anti-rabbit IgG	CW0103S	Cwbio	Beijing, China	1 : 3000

## Data Availability

All data generated or analyzed during this study are included in this published article.
